# Supplementary material for: Effects of non-technical skill-based surgical education for trainees on herniorrhaphy outcomes
Source: PLoS One. 2023 Sep 19;18(9):e0291698. doi: 10.1371/journal.pone.0291698 (PMC10508599; doi:10.1371/journal.pone.0291698)
Supplement: Attachment — Submitted filename: dr. hazim alhiti Review Report 713.doc [file pone.0291698.s002.doc]

**This is the review report of Dr. Hazim Abdul Rahman Alhiti**

**I The Information of** Research Paper Review

| **Paper’s File Name** | PONE-D-23-14386 | | | | |
| --- | --- | --- | --- | --- | --- |
| **Journal Title** | PLOS ONE | | | | |
| **Paper Title** | Effects of non-technical skill-based surgical education for trainees on herniorrhaphy outcomes | | | | |
| **Date received** | 20-6-2023 | | | | |
| **Date reviewed** | 22-6-2023 | | | | |
| **Reviewer’s Name** | **HAZIM ABDUL RAHMAN ALHITI** | | | | |
| **Affiliation** | **Dr. Hazim Abdul Rahman Alhiti**  **General Surgeon Specialist M.D**  **Al-Ramadi Teaching Hospital**  **Chief of Breast Clinic (Hit General Hospital)**  **Chief of Cancer Clinic (Hit General Hospital)**  **General surgeon specialist in Alrahma private Hospital- Baghdad**  **Academic lecturer in al Mansoor university college-Baghdad**  **Academic lecturer in college of medicine-university of Fallujah**  **Academic lecturer in college of medicine-Al Iraqia University**  **Academic lecturer (Electronic learning)- Tikrit University**  **Academic external examiner-college of medicine- University of Baghdad**  **Academic external examiner-college of medicine- University of Ibn Sina**  **Academic external examiner- college of medicine-University of Al-Qadisiyah**  **Academic lecturer in Higher Institute of Health, Al Anbar Directorate of Health**  **head of the department in The Middle East university college-Baghdad**  **Head of emergency department, Higher Institute of Health, Al Anbar Directorate of Health**  **Author, Reviewer and editor**  **009647821490455**  **009647509605075**  [**hazim4436@gmail.com**](mailto:hazim4436@gmail.com)  [**dr.hazim.abdul@meuc.edu.iq**](mailto:dr.hazim.abdul@meuc.edu.iq)  [**hazim.alhiti@uoanbar.edu.iq**](mailto:hazim.alhiti@uoanbar.edu.iq)  [**hazim.abdulrahman@uofallujah.edu.iq**](mailto:hazim.abdulrahman@uofallujah.edu.iq)  [**hazim.extpht@muc.edu.iq**](mailto:hazim.extpht@muc.edu.iq)  [**https://www.webofscience.com/wos/author/record/V-5796-2018**](https://www.webofscience.com/wos/author/record/V-5796-2018)  [**https://orcid.org/0000-0003-0000-8267**](https://orcid.org/0000-0003-0000-8267)  [**https://www.researchgate.net/profile/Hazim-Alhiti**](https://www.researchgate.net/profile/Hazim-Alhiti)  [**https://iraqiboard.academia.edu/TheHazim**](https://iraqiboard.academia.edu/TheHazim)  [**https://www.linkedin.com/in/hazim-alhiti-315607186/**](https://www.linkedin.com/in/hazim-alhiti-315607186/) | | | | |
| **General comments** | | | | | |
| 1. **the main content of research presented in the paper is** Effects of non-technical skill-based surgical education for trainees on herniorrhaphy outcomes 2. **the main proposed paper is to** evaluate the learning curve for non-technical skillbased education in herniorrhaphy 3. **the deduced conclusions based on the research methods/cases are** . An early decline and deep plateau of the learning curve can be achieved with well-implemented quality improvement initiatives. 4. **the conclusions are tenable** 5. **the progress of this paper is good compared with the current research results** | | | | | |
| **Strengths and weaknesses** | | | | | |
| 1. **the topic is not unique but worthy of researching** 2. **there are hundreds of papers in google scholar and Refseek about this topic since 2019** 3. **the title is attractive** 4. **the abstract is informative** 5. **the aim is clear** 6. **the KEYWORDS are good** 7. **lack of the abbreviations section** 8. **the introduction provide sufficient background information for readers in the immediate field to understand the problem/hypotheses** 9. **the text arrangement is good** 10. **the method section is clear** 11. **the depth of the academic material is good** 12. **the study design is good** 13. **The suitability and accuracy of questions is good** 14. **The research methodology is clear** 15. **The materials are suitable** 16. **the logic is clear** 17. **the paper is not novel** 18. **there are few grammatical errors in this article** 19. **the related concepts are introduced** 20. **the readability is sufficient** 21. **the results are good** 22. **all figures/tables are clear enough to summarize the results for presentation to the readers** 23. **all figures/tables are well referred to in the text** 24. **the theoretical analysis in this article is sufficient** 25. **the discussion of results from multiple angles is sufficient** 26. **the conclusion is good** 27. **the reference section contains too many old ref** 28. **please use (google scholar and Refseek) search engines then set it since 2019** 29. **the references are in order within the text** 30. **Bias is present** 31. **There is no conflict of interest with the author about this topic** 32. **Fund is mentioned** 33. **Ethical approval is mentioned** 34. **Conflict of interest** **is mentioned** 35. **Acknowledgement** **is mentioned** 36. **You can use my suggestions**   **My final decision is acceptable after minor revision** | | | | | |
| **Paper score** | | | | | |
| **Ten-point System** | **Relevance** | **Originality** | **Significance** | **Technical soundness** | **Clarity of presentation / language** |
| **Enter a score between 0** **to 10** | **8** | **8** | **9** | **9** | **9** |
| **Final Evaluation** | | | | | |
| **☐ Excellent** **☐ Very Good** **☐ Good** **☐ Fair** **☐ Poor** | | | | | |
| **☐ Acceptance** **☐ Minor Revision** **☐ Major Revision** **☐ Rejection** | | | | | |

| **Part No.** | **Description of the Part of Manuscript** | **Score**  **0 to 10** |
| --- | --- | --- |
| 1. | **Title**  a. Accurate | 9 |
|  | b. Effective | 9 |
|  | c. Represents Main Message of Paper | 9 |
| 2. | **ABSTRACT**  a. Size of abstract (maximum 250 words) | 9 |
|  | b. Objective | 9 |
|  | c. Methodology | 9 |
|  | d. Results | 9 |
|  | e. Conclusion | 9 |
|  | f. Key Words | 9 |
| 3. | difficult words/key words if relevant | 0 |
| 4. | **introduction**  a. It start Relevant | 9 |
|  | b. Subject of Paper Made Clear | 9 |
|  | c. Importance of Study Made Clear | 9 |
|  | d. Reasons for the Study Stated | 9 |
|  | e. Significant finding of the Study Stated | 9 |
|  | f. Available International, National, Ancient Literature Recorded with  References of at least 05 years (60%) and in Vancouver style | 9 |
|  | g. References appropriate for Discussion are not to be mentioned in  Introduction | 9 |
| 5 | **Aims and objectives of the study**  a. Are clearly Stated | 9 |
|  | b. Are relevant with study | 9 |
| 6 | **methodology**  a. Population of uniform standard | 9 |
|  | b. Sample selection criteria reliable | 9 |
|  | c. Inclusion/Exclusion criteria specified | 9 |
|  | d. Control within the study or literature given | 9 |
|  | e. Important Variable Measurement Criteria specified | 9 |
|  | f. Investigation/Procedure/Technique clearly given | 9 |
| 7 | **Results and analysis of results**  a. Presentation of results clear | 9 |
|  | b. Data relevant to objectives of the study | 9 |
|  | c. All subjects/Variable in the study accounted for | 9 |
|  | d. Results reported the drop out (if present) | 9 |
|  | e. Results presented in appropriately | 9 |
|  | f. Results presented with standard deviation | 9 |
|  | g. Appropriate statistical tests applied | 9 |
|  | h. Data Analysis correctly interpreted | 9 |
|  | i. Level of significance (preferred less than 0.5 %) |  |
| 8 | **Discussion**  a. Important findings and results discussed | 9 |
|  | b. New emerging findings discussed | 9 |
|  | c. Results of the study compared/discussed with other past studies | 9 |
|  | d. Results and analysis leads to firm conclusions | 9 |
| 9 | **Conclusions**  a. Conclusion is in accordance with data analysis and results | 9 |
|  | b. Conclusions relevant to the aims and objectives of the study | 9 |
|  | c. Conclusions based on critical argument | 9 |
|  | d. Future recommendations if return | 9 |
| 10 | **WRITTEN english**  a. Spelling | 9 |
|  | b. Grammar | 9 |

Length of the paper:

|  | **Should be extended** | **Perfect** | **Should be shortened** |
| --- | --- | --- | --- |
| **ABSTRACT** |  | **X** |  |
| **PAPER** |  | **X** |  |

**This paper should be published as:**

| **Regular paper** | **X** |
| --- | --- |
| **Short paper** |  |
| **Commentary Paper** |  |

**This paper should be published as:**

| **TOP TOPIC IN THE JOURNAL** |  |
| --- | --- |
| **GOOD TOPIC IN THE JOURNAL** | **X** |
| **FAIR TOPIC IN THE JOURNAL** |  |

Types of bias:

| **Follow up bias** |  |
| --- | --- |
| **Exclusion bias** |  |
| **Analysis bias** |  |
| **Reporting bias** | **X** |
| **Assembly bias** |  |
| **Measurement bias** |  |
| **Detection bias** | **X** |
| **Selection (susceptibility) bias** | **X** |
| **Chronology bias** |  |
| **Channeling bias** |  |
| **Response bias** |  |

Types Conflicts of interests:

| **Between professionals** |  |
| --- | --- |
| **Competing loyalties** |  |
| **Relationship conflicts** |  |
| **Self-dealing** |  |
| **Structural conflicts** |  |
| **Communication** |  |
| **Data conflicts** |  |
| **personal** |  |
| **Gifts** |  |
| **Subject related** |  |
| **Interest conflicts** |  |
| **Value conflicts** |  |

| **Overall recommendation** | **YES** |
| --- | --- |
| Acceptable without any changes |  |
| Acceptable after minor modifications | **X** |
| Major revision: needs to be re-written and re-evaluated |  |
| Unacceptable |  |

**Questions to the authors:**

1. **You can use my suggestions**

**To the editor:**

**Dear Editor, thank you for giving me an opportunity to review this manuscript**

**This is an expert author**

**The paper needs a specialist for statistics.**

**Please check the plagiarism**

**my final decision is acceptable after minor revision**

**Date: 22/6 / 2023**
